# Non-Lethal Doses of RSL3 Impair Microvascular Endothelial Barrier through Degradation of Sphingosie-1-Phosphate Receptor 1 and Cytoskeletal Arrangement in A Ferroptosis-Independent Manner

**DOI:** 10.3390/biomedicines11092451

**Published:** 2023-09-04

**Authors:** Boina Baoyinna, Jiaxing Miao, Patrick J. Oliver, Qinmao Ye, Nargis Shaheen, Timothy Kalin, Jinshan He, Narasimham L. Parinandi, Yutong Zhao, Jing Zhao

**Affiliations:** 1 Department of Physiology and Cell Biology, Dorothy M. Davis Heart and Lung Research Institute, The Ohio State University, Columbus, OH 43210, USA; 2 Department of Internal Medicine, The Ohio State University, Columbus, OH 43210, USA

**Keywords:** RSL3, S1PR1, endothelial permeability, cytoskeletal arrangement, protein degradation

## Abstract

The excess microvascular endothelial permeability is a hallmark of acute inflammatory diseases. Maintenance of microvascular integrity is critical to preventing leakage of vascular components into the surrounding tissues. Sphingosine-1-phosphate (S1P) is an active lysophospholipid that enhances the endothelial cell (EC) barrier via activation of its receptor S1PR1. Here, we delineate the effect of non-lethal doses of RSL3, an inhibitor of glutathione peroxidase 4 (GPX4), on EC barrier function. Low doses of RSL3 (50–100 nM) attenuated S1P-induced human lung microvascular barrier enhancement and the phosphorylation of AKT. To investigate the molecular mechanisms by which RSL3 attenuates S1P’s effect, we examined the S1PR1 levels. RSL3 treatment reduced S1PR1 levels in 1 h, whereas the effect was attenuated by the proteasome and lysosome inhibitors as well as a lipid raft inhibitor. Immunofluorescence staining showed that RSL3 induced S1PR1 internalization from the plasma membrane into the cytoplasm. Furthermore, we found that RSL3 (100 and 200 nM) increased EC barrier permeability and cytoskeletal rearrangement without altering cell viability. Taken together, our data delineates that non-lethal doses of RSL3 impair EC barrier function via two mechanisms. RSL3 attenuates S1P1-induced EC barrier enhancement and disrupts EC barrier integrity through the generation of 4-hydroxynonena (4HNE). All these effects are independent of ferroptosis.

## 1. Introduction

Endothelial cells form a continuing monolayer that constitutes the inner cellular lining of vascular vessels. Macromolecules and blood cells in the flowing blood are prevented from leaking into surrounding tissue by the confluent endothelial barrier, which acts as the gatekeeper of vascular homeostasis [1,2,3]. In the setting of acute inflammatory disease, microvascular endothelial permeability is abnormally increased, causing nearby tissue edema. This excess microvascular endothelial permeability is a hallmark of acute inflammatory disorders, such as acute lung injury [4,5,6,7,8]. Cytoskeletal rearrangement under inflammatory stimuli contributes to disruption of vascular barrier integrity [7,8,9,10].

Ferroptosis is a form of iron-dependent cell death that is characterized by oxidative damage and excess lipid peroxidation [11,12,13]. Inducing ferroptosis in a wide range of cancer cells has been shown to play a pivotal role in tumor suppression [14,15,16]. Vascular ECs undergo ferroptosis in various vascular diseases [17,18]. RAS-selective lethal 3 (RSL3) is an inhibitor of GPX4 and has been well used as an inducer of ferroptosis [19,20] and is a potential drug candidate to treat cancers. RSL3 binds and inactivates GPX4, thereby causing lipid peroxidation, membrane damage, and ferroptosis [19,20]. The challenges of development of small molecules as anti-tumor drugs are causing non-cancer cell damage including ECs. The minimal dose of RSL3 for inducing ferroptosis of cancer cells is 0.4 μM [21]. RSL3 treatment (over 0.5 μM) for 24 h induced cell death in ECs [22]. However, the effect of non-lethal doses of RSL3 on ECs has not been reported. Here we investigate the effect of low doses of RSL3 (0.05–0.2 μM) on EC barrier function without altering cell viability.

S1P is a product of the metabolism of sphingosine lipids. Sphingosine kinases catalyze phosphorylation of sphingosine and generate S1P [23,24]. The bioactivity of S1P occurs through ligation to its G-protein-coupled receptors, S1PRs [25,26,27,28]. Enhancement of the EC barrier by S1P has been well documented. S1P activates S1PR1 to trigger signaling pathways, including the phosphorylation of AKT and activation of Rac1, resulting in increased EC barrier integrity [27,29,30,31]. S1PR1 is localized to lipid rafts of the plasma membrane of ECs. S1PR1 internalization and degradation has been studied. Oo, M.L. et al. have shown that S1PR1 is degraded by the ubiquitin-proteasome system [32]. The role of RSL3 in S1PR1 degradation has not been reported. 

Cytoskeletal rearrangement is a major mechanism of EC barrier disruption. Phosphorylation of myosin light chains (MLC) results in the contraction of actomyosin and the formation of stress fibers [33,34,35]. MLC phosphorylation is mediated by activation of kinases including MLC kinase, RhoK, and p38 MAPK [36,37]. The effect of RSL3 on cytoskeletal rearrangement has not yet been revealed. In this study, we reveal that at non-lethal concentrations, RSL3 modulates EC barrier function through both degrading S1PR1 and increasing cytoskeletal rearrangement. 

## 2. Materials and Methods

Cell culture and reagents: Human lung microvascular endothelial cells (HLMVECs, from Lonza, Walkersville, MD, USA) were cultured with endothelial growth medium. A cell line of human lung microvascular endothelial cells (HULEC-5a, SV40 large T antigen transformed, from ATCC, Manassas, VA, USA) was cultured in Dulbecco’s Modified Eagle Medium (DMEM) with 10% fetal bovine serum (FBS). HEK293/TLR4-MD2-CD14 (HEK293) cells (from InvivoGen, San Diego, CA, USA) were cultured in DMEM with 10% FBS and antibiotics. Antibodies against Flag tag, RSL3, erastin, 4HNE, N-acetylcysteine (NAC), MLN7243, methyl-β-cyclodextrin (MBCD), cycloheximide (CHX), dipeptidyl peptidase IV inhibitor (DDP4-M), and S1P were purchased from Sigma (St. Louis, MO, USA). Antibodies against phospho (p)-AKT, AKT, p-p38 MAPK, p38 MAPK, p-MLC, MLC, and ubiquitin were purchased from Cell Signaling (Danvers, MA, USA). Antibodies against S1PR1, immobilized protein A/G beads, and control IgG were purchased from Santa Cruz Biotechnology (Sant Cruz, CA, USA). Antibodies against 4HNE were purchased from Thermo Fisher Scientific (Waltham, MA, USA). MG132 and leupeptin were purchased from ENZO Life Sciences (Farmingdale, NY, USA). All materials used in the experiments are of the highest grade commercially available. 

Electrical cell-substrate impedance sensing system (ECIS): HLMVECs and HULEC-5a cells grown (100% confluency) in a chamber with gold electrodes (8W10E+, Applied Biophysics, Troy, NY, USA) at 37 °C with 5% CO_2_ were treated with RSL3 or S1P as indicated, and transendothelial electrical resistances (TEER) were acquired at 4000 Hz in real time using an ECIS Zθ (Applied Biophysics, Troy, NY, USA) system. TEER values from each microelectrode were pooled at discrete time points and plotted as the mean ± SEM [38]. In a wound healing assay, HLMVECs grown (100% confluency) on 8W1E ECIS plates at 37 °C with 5% CO_2_ were treated with a wounding current of 3000 µA at 60 kHz for 30 s, and cells were then treated with RSL3 (50 and 100 nM). Wound healing was measured by changes in TEER. 

Plasmid transfection: Human S1PR1 cDNA with N terminal Flag tag in pCMV3 plasmid was purchased from Sino Biological Inc., Wayne, PA, USA). LipoJet plasmid transfection reagent (SignaGen Laboratories, Frederick, MD, USA) was used for transfecting HULEC-5a and HEK293 cells in 6-well culture plates according to the manufacturer’s instructions. Briefly, 1 μg plasmid was mixed with 3 μL LipoJet Reagent in 200 μL diluted transfection buffer at room temperature for 10 min, and then the LipoJet/plasmid mix was added in serum containing medium. Cells were collected and analyzed after 48 h of transfection. 

Immunoblotting and immunoprecipitation (IP): After treatment, cells were washed with cold phosphate-buffered saline (PBS) and then lysed in lysis buffer containing 2-mercaptoethanl (Cell signaling, Danvers, MA). 20 μg of total proteins were separated in SDS-PAGE gels, transferred to nitrocellulose membrane, and then immunoblotted with certain primary and secondary antibodies sequentially. For IP, 1 mg of denatured cell lysates was incubated with a primary antibody overnight at 4 °C, followed by incubation with protein A/G beads for an additional 2 h. The beads were rinsed with PBS and lysis buffer 3 times. Proteins on the beads were eluted in 2× SDS sample buffer and analyzed via immunoblotting [39]. Protein bands were detected using the Enhanced Chemiluminescence Detection Kit (Thermo Fisher Scientific, USA) in the Azure C600 Image System (VWR International, Radnor, PA, USA). 

qRT-PCR analysis: After RSL3 treatment (50 nM, 1 h), HULEC-5a cells were collected. RNA extraction was performed using a total RNA extraction kit (Invitrogen) according to the manufacturer’s instructions. The expression of *S1PR1* was performed using iQ SYBR Green Supermix and the iCycler real-time PCR detection system (Bio-Rad, Hercules, CA, USA). *GAPDH* was used as an internal control. The sequences of specific primer pairs are described below: *S1PR1* forward, GGCTCTCCGAACGCAACTTC, *S1PR1* reverse, GTTCGATGAGTGATCCAGGCT; h*GAPDH* forward, TCGGAGTCAACGGATTTGGTCG, h*GAPDH* reverse, GCTCTCCAGAACATCATCCCTGCCT-3.

Immunofluorescence staining: HEK293, HLMVECs, and HULEC-5a cells were cultured in glass-bottom dishes (MatTek, Ashland, MA, USA). Cells were transfected or treated as indicated. After being washed with PBS, cells were fixed with 3.7% formaldehyde in TBST buffer for 20 min. Cells were blocked by 1% BSA in TBST, followed by incubation with primary antibodies for 1 h and sequentially with fluorescence-labeled secondary antibodies and DAPI for an additional 1 h. Immunofluorescent cell imaging was captured using a Nikon A1R confocal microscope. 

Statistical analysis—Immunoblots were quantified with Image J and normalized to intensities of internal control immunoblots. Statistical analysis was performed using GraphPad Prism software, version 8.0 (GraphPad Software, San Diego, CA, USA). All results were subjected to two-way ANOVA and, wherever appropriate, Student’s *t*-test. Data are expressed as mean ± SEM of triplicate samples from at least three independent experiments, and *p* values < 0.05 were considered statistically significant.

## 3. Results

### 3.1. RSL3 Attenuates S1P-Induced EC Barrier Enhancement and Phosphorylation of AKT 

**Figure 1:**
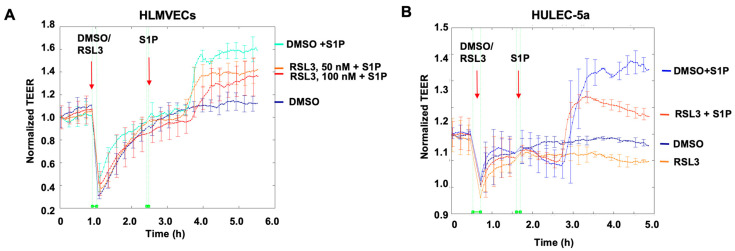
RSL3 attenuates S1P-induced EC barrier enhancement. (**A**) HLMVECs were pretreated with RSL3 (0, 50, and 100 nM) for 1 h, followed by treatment with S1P (1 μM). TEER at 4000 Hz was measured by the ECIS system. Each line represents the mean ± SEM at the specified time points. (**B**) HULEC-5a cells were pretreated with RSL3 (0 and 50 nM) for 1 h, followed by treatment with S1P (1 μM). TEER at 4000 Hz was measured by the ECIS system. Each line represents the mean ± SEM at the specified time points.

**Figure 2:**
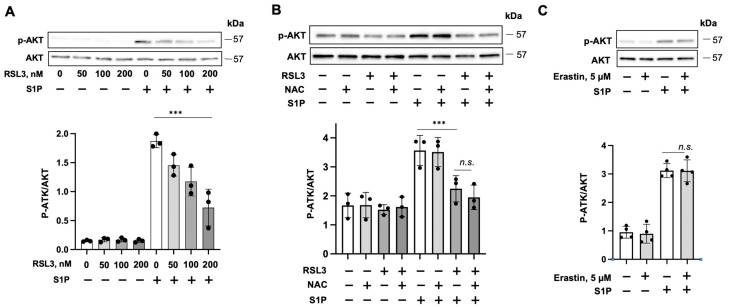
RSL3 attenuates S1P-induced phosphorylation of AKT. (**A**) HULEC-5a cells were pretreated with RSL3 (0, 50, 100, and 200 nM) for 1 h, followed by treatment with S1P (1 μM) for an additional 15 min. Cell lysates were analyzed via immunoblotting with phosphor (p)-AKT and AKT antibodies. Quantification of p-AKT protein levels relative to AKT were assessed by densitometry (*n* = 3). Significant differences (*** *p* < 0.001) between the two groups as determined using two-way ANOVA. (**B**) HULEC-5a cells were treated with or without NAC (10 mM) for 30 min, followed by treatment with RSL3 (50 nM) for 1 h. Cells were then treated with S1P (1 μM) for an additional 15 min. Cell lysates were analyzed via immunoblotting with p-AKT and AKT antibodies. Quantification of p-AKT protein levels relative to AKT were assessed using densitometry (*n* = 3). Significant differences (*** *p* < 0.001) or no significance differences (*p* > 0.05, n.s.) between the two groups as determined using two-way ANOVA. (**C**) HULEC-5a cells were treated with or without erastin (5 μM) for 1 h followed by treatment with S1P (1 μM) for an additional 15 min. Cell lysates were analyzed using immunoblotting with p-AKT and AKT antibodies. Quantification of p-AKT protein levels relative to AKT was assessed using densitometry (*n* = 4). No significant differences (*p* > 0.05, n.s.) between the two groups as determined by two-way ANOVA. Immunoblots are representatives of three to six independent experiments.

RSL3-induced ferroptosis in ECs has been reported [17,18]; however, the effect of non-lethal doses of RSL3 on EC function has not been studied. S1P has been shown to enhance EC barrier integrity [27,29,30,31]. First, we examined the effect of RSL3 on S1P-induced EC barrier function by using an ECIS system. HLMVECs were cultured in ECIS chambers until reaching confluence. Cells were treated with either DMSO or RSL3 (50 and 100 nM) for 1 h, followed by the addition of 1 μM S1P. S1P induced increases in TEER, suggesting that S1P promotes the EC barrier, while pretreatment with RSL3 attenuated the effect (Figure 1A). This was confirmed in human lung microvascular cell line HULEC-5a cells (Figure 1B). AKT is a downstream signal protein of S1P receptors. To investigate if the effect of RSL3 on S1P-induced EC barrier enhancement occurs through modulation of S1P-mediated signaling, we examined the phosphorylation of AKT. S1P treatment for 15 min induced phosphorylation of AKT, while the effect was attenuated by pretreatment with RSL3 in a dose-dependent manner (Figure 2A). RSL3 at lethal concentration has been shown to induce ROS accumulation. We examined if reactive oxygen species (ROS) regulate RSL3-mediated S1P signaling. As shown in Figure 2B, NAC, a free radical scavenger, had no effect on RSL3-attenuated phosphorylation of AKT by S1P, suggesting that ROS does not contribute to the effect of RSL3. Further, we found that pretreatment with another ferroptosis activator, erastin, had no effect on S1P-induced phosphorylation of AKT (Figure 2C), suggesting that the effect of RSL3 on S1P-mediated AKT activation is independent of ferroptosis signaling. 

### 3.2. RSL3 Induces S1PR1 Degradation and Internalization

**Figure 3:**
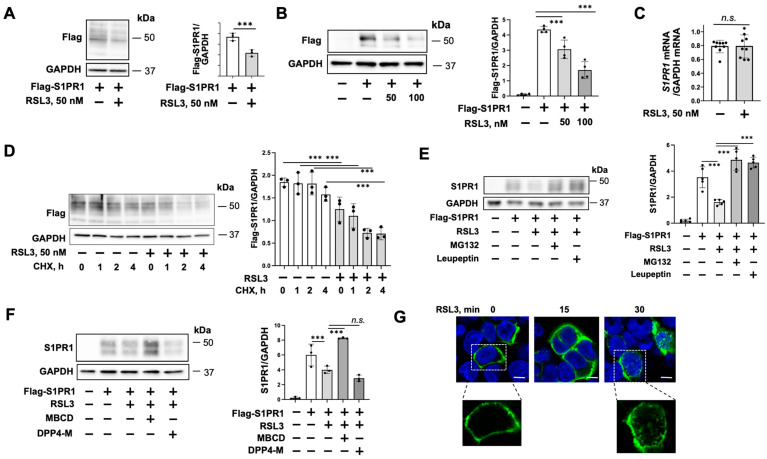
RSL3 induces S1PR1 degradation and internalization, and the effects were attenuated by MG132, leupeptin, and MBCD. (**A**) HULEC-5a cells were transfected with Flag-S1PR1 plasmid for 48 h and then treated with RSL3 (50 nM) for 1 h. Immunoblotting analysis was performed with indicated antibodies. Quantification of Flag-S1PR1 protein levels relative to GAPDH was assessed by densitometry (*n* = 3). Significant differences (*** *p* < 0.001) between the two groups were determined via two-way ANOVA. (**B**) HEK293 cells were transfected with Flag-S1PR1 plasmid for 48 h and then treated with RSL3 (50 and 100 nM) for 1 h. Immunoblotting analysis was performed with indicated antibodies. Quantification of Flag-S1PR1 protein levels relative to GAPDH was assessed using densitometry (*n* = 4). Significant differences (*** *p* < 0.001) between the two groups were determined using two-way ANOVA. (**C**) HULEC-5a cells were treated with RSL3 (50 nM) for 1 h. S1PR1 mRNA levels were examined using RT real-time PCR. S1PR1 mRNA levels were normalized to GAPDH mRNA levels. No significant differences (*p* > 0.1, n.s.) between the two groups were determined using two-way ANOVA. (**D**) HULEC-5a cells were transfected with Flag-S1PR1 plasmid for 48 h and then treated with RSL3 (50 nM) for 1 h, followed by CHX (20 μg/mL) treatment for additional 1, 2, and 4 h. Immunoblotting analysis was performed with indicated antibodies. Quantification of Flag-S1PR1 protein levels relative to GAPDH were assessed by densitometry (*n* = 3). Significant differences (*** *p* < 0.001) were determined using two-way ANOVA. (**E**,**F**) HEK293 cells were transfected with Flag-S1PR1 plasmid for 48 h and then treated with MG-132 (20 μM), leupeptin (100 μM) (**E**), MBCD (10 μM), and DDP4-M (10 μM) (**F**), followed by RSL3 (50 nM) for 1 h. Immunoblotting analysis was performed with indicated antibodies. Quantification of Flag-S1PR1 protein levels relative to GAPDH were assessed using densitometry (*n* = 3–5). Significant differences (*** *p* < 0.001) between the two groups were determined by two-way ANOVA. Immunoblots are representatives of three independent experiments. (**G**) HEK293 cells grown on glass-bottom dishes were transfected with Flag-S1PR1 plasmid for 48 h and then treated with RSL3 (50 nM) for 15 and 30 min. Cells were immunofluorescence stained with a Flag antibody (green). Nuclei were stained with DAPI (blue). Scale bars = 5 μm.

The EC barrier protective effect of S1P occurs through the activation of S1PR1 [27,29,30,31]. We examined if RSL3 modulates S1PR1 levels. Flag-S1PR1 was overexpressed in HULEC-5a cells and then treated with RSL3 (50 nM) for 1 h. RSL3 significantly downregulated S1PR1 levels (Figure 3A). The data was confirmed in RSL3-treated HEK293 cells (Figure 3B). To examine the molecular mechanisms by which RSL3 reduces S1PR1 levels, we examined the mRNA levels of S1PR1. As shown in Figure 3C, RSL3 had no effect on S1PR1 mRNA levels. Furthermore, CHX chase assay showed that RSL3 pretreatment reduced S1PR1 half-life (Figure 3D), suggesting that the effect of RSL3 on S1P-mediated EC function and intracellular signaling occurs through promoting S1PR1 degradation. It has been shown that S1PR1 turnover is mediated by the ubiquitin–proteasome system. We examined the effect of inhibition of proteasomes and lysosomes on RSL3-induced S1PR1 degradation. The inhibitors of proteasomes (MG132) and lysosomes (leupeptin) both attenuated RSL3-induced S1PR1 degradation (Figure 3E). Dipeptidylpeptidase IV inhibitor had no effect on S1PR1 levels (Figure 3F). Lipid rafts on the plasma membrane are platforms for S1PR1 activation and internalization [40]. Disruption of lipid rafts by MBCD prevented S1PR1 degradation (Figure 3F). Furthermore, immunofluorescence staining revealed that S1PR1 is localized on the plasma membrane in the DMSO-treated control HEK293 cells. RSL3 treatment for 30 min induced S1PR1 internalization into the cytoplasm (Figure 3G). Taken together, RSL3 induces S1PR1 degradation and internalization.

### 3.3. Determination of Molecular Mechanisms by Which RSL3 Induces S1PR1 Degradation

**Figure 4:**
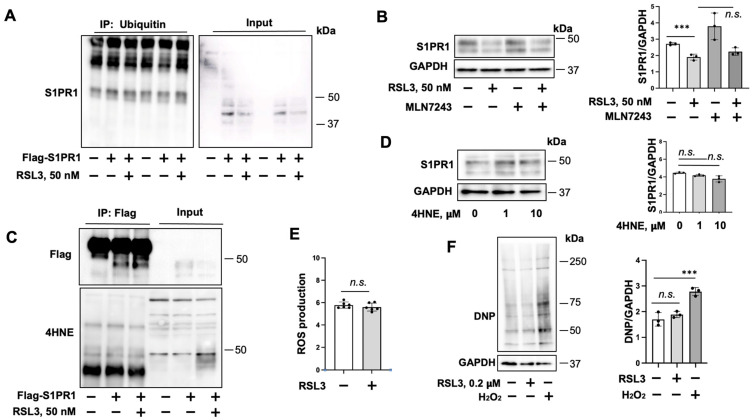
RSL3-induced S1PR1 degradation is not regulated by ubiquitination and oxidation. (**A**) HEK293 cells were transfected with Flag-S1PR1 and HA-ubiquitin plasmids for 48 h, and then cells were treated with RSL3 (50 nM) with MG132 (20 μM) for 1 h. Denatured cell lysates were subjected to ubiquitination assay by IP Flag, followed by ubiquitin immunoblotting. (**B**) HEK293 cells were transfected with Flag-S1PR1 plasmid for 48 h. Cells were pretreated with MLN7243 (10 μM) for 0.5 h, and then cells were treated with RSL3 (50 nM) for an additional 1 h. Cell lysates were subjected to immunoblotting with antibodies against S1PR1 and GAPDH. Quantification of S1PR1 protein levels relative to GAPDH were assessed via densitometry (*n* = 3). Significant differences (*** *p* < 0.001) or no significant (n.s.) differences (*p* > 0.1) between the two groups was determined using two-way ANOVA. (**C**) HEK293 cells were transfected with Flag-S1PR1 plasmid for 48 h, and then cells were treated with RSL3 (50 nM) with MG132 (20 μM) for 1 h. Denatured cell lysates were subjected to IP Flag, followed by Flag and 4HNE immunoblotting. (**D**) HEK293 cells were transfected with Flag-S1PR1 plasmid for 48 h. Cells were treated with 4HNE (1 and 10 μM) for 1 h. Cell lysates were subjected to immunoblotting with antibodies against S1PR1 and GAPDH. Quantification of S1PR1 protein levels relative to GAPDH were assessed by densitometry (*n* = 3). No significant (n.s.) differences (*p* > 0.1) between the two groups was determined using two-way ANOVA. (**E**) HULEC-5a cells were treated with RSL3 (50 nM) for 1 h. ROS production was determined via DCFDA assay. N = 6, no significant (n.s.) differences (*p* > 0.1) between the two groups were determined via two-way ANOVA. (**F**) HULEC-5a cells were treated with RSL3 (50 nM) or H_2_O_2_ (0.4 mM) for 1 h. Protein oxidation assay was performed using OxyBlot protein oxidation detection kit (Millipore). N = 3, significant differences (*** *p* < 0.001) or no significant (n.s.) differences (*p* > 0.1) between the two groups were determined by two-way ANOVA. Immunoblots are representatives of two (**A**,**C**) or three (**B**,**D**,**F**) independent experiments.

S1PR1 ubiquitination triggers its degradation [32]. To investigate if RSL3 induces S1PR1 ubiquitination, we performed an in vivo ubiquitination assay. Ubiquitination of S1PR1 was not detected after RSL3 treatment (Figure 4A). Inhibition of E1 activating enzyme by MLN7243 did not rescue RSL3-induced S1PR1 degradation (Figure 4B), suggesting that ubiquitination is not involved in the effect of RSL3 on S1PR1 degradation. We found that RSL3 increased 4HNE in 1 h; however, IP showed no 4HNE modification on S1PR1 (Figure 4C). Treatment with 4HNE (1 and 10 μM) did not alter S1PR1 levels (Figure 4D). Surprisingly, ROS production (Figure 4E) and protein oxidation (Figure 4F) were not increased in response to RSL3 (50 nM, 1 h). This might be due to the low level of ROS generated by the low concentration of RSL3. Taken together, RSL3-induced S1PR1 degradation is not dependent on ubiquitination and protein oxidation. Based on the effects of MG132, leupeptin, and MBCD shown in Figure 3, it is possible that RSL3-induced S1PR1 degradation occurs through regulating its internalization and its proteasomal and lysosomal degradation.

### 3.4. Relatively Higher Concentration of RSL3 Increases EC Permeability

**Figure 5:**
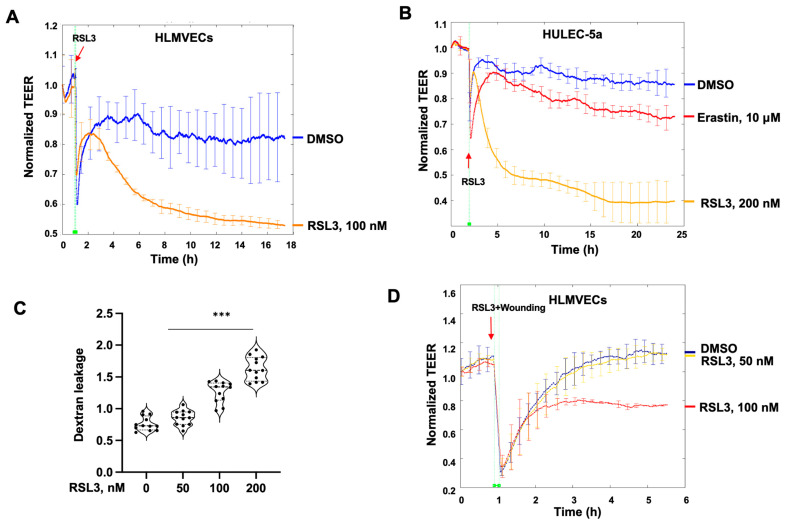
RSL3 (100 and 200 nM) increases EC permeability and attenuates EC wound healing. (**A**) HLMVECs were treated with RSL3 (100 nM), and then TEER at 4000 Hz was measured by the ECIS system. Each line represents the mean ± SEM at the specified time points. (**B**) HULEC-5a cells were treated with RSL3 (100 nM) or Erastin (10 μM), and then TEER at 4000 Hz was measured by the ECIS system. Each line represents the mean ± SEM at the specified time points. (**C**) HULEC-5a cells were cultured on Transwell plate inserts with a pore size of 0.4 μm. Cells were treated with RSL3 (0, 50, 100, and 200 nM) for 1 h. FITC-labeled dextran (40 kDa) leakage from top to bottom was measured (*n* = 12). Significant differences (*** *p* < 0.001) between the two groups were determined via two-way ANOVA. (**D**) HLMVECs grown on 8W1E ECIS plates and a wounding current of 3000 μA at 60 kHz was delivered for 30 s. Cells were treated with RSL3 (50 and 100 nM). Cell migration was measured as changes of TEER in the ECIS system.

RSL3 alone at 50 nM did not significantly change EC permeability (Figure 1B), while RSL3 at 100 and 200 nM reduced TEER (Figure 5A,B), suggesting that relatively higher concentrations of RSL3 increases EC permeability. Erastin, another ferroptosis activator, slightly reduced TEER, but the effect was largely less than RSL3 (Figure 5B). Further, a Transwell leakage assay was used to measure HULEC-5a monolayer permeability. RSL3 at 100–200 nM increased dextran leakage (Figure 5C), supporting the data obtained from ECIS. Microvascular EC migration plays a critical role in vascular repair and remodeling after tissue injury. To determine if RSL3 affects EC migration, we performed a wound healing assay using ECIS. Control cells (DMSO-treated) and 50 nM RSL3-treated cells filled the wound area in 4–5 h after wounding, while 100 nM RSL3-treated cells significantly reduced cell migration and wound healing (Figure 5D), suggesting that RSL3 at above 100 nM significantly impacts ECs by increasing permeability and decreasing cell migration.

### 3.5. RSL3 Increases Stress Fibers and Gap Formation in ECs through Increasing 4HNE

**Figure 6:**
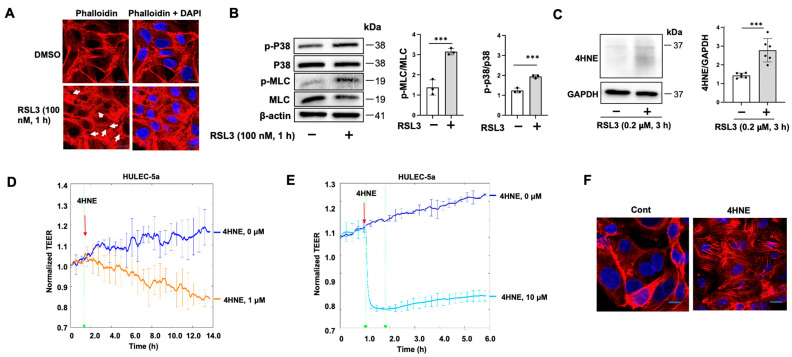
RSL3 (100 nM) induces cytoskeletal rearrangement. (**A**) HLMVECs grown on glass-bottom dishes were treated with RSL3 (100 nM) for 1 h. Stress fiber formation was examined via phalloidin staining (red). Nuclei were stained with DAPI (blue). Scale bars = 10 μm. Arrows indicate gaps. (**B**) HLMVECs were treated with RSL3 (100 nM) for 1 h, and then cell lysates were subjected to immunoblotting analysis with indicated antibodies. Quantification of p-MLC or p-p38 MAPK protein levels relative to MLC or p38 MAPK were assessed by densitometry (*n* = 3). Significant differences (*** *p* < 0.001) between the two groups were determined by two-way ANOVA. (**C**) HULEC-5a cells were treated with RSL3 (200 nM) for 3 h. Cell lysates were subjected to 4HNE immunoblotting. Quantification of 4HNE levels relative to GAPDH were assessed using densitometry (*n* = 6). Significant differences (*** *p* < 0.001) between the two groups were determined via two-way ANOVA. Immunoblots are representatives of three to six independent experiments. (**D**,**E**) HULEC-5a cells were treated with 4HNE (1 and 10 μM), and then TEER at 4000 Hz was measured by the ECIS system. Each line represents the mean ± SEM at the specified time points. (**F**) HULEC-5a grown on glass-bottom dishes were treated with 4HNE (5 μM) for 2 h. Stress fiber formation was examined via phalloidin staining (red). Nuclei were stained with DAPI (blue). Scale bars = 10 μm.

Increases in stress fibers and gap formation are the major mechanisms of EC monolayer barrier disruption [7]. Not surprisingly, RSL3 (100 nM, 1 h) increased stress fibers and gap formation (Figure 6A). Furthermore, increases in phosphorylation of MLC and p38 MAPK were detected in RSL3 (100 nM, 1 h)-treated HULEC-5a cells (Figure 6B), suggesting that RSL3 regulates cytoskeletal rearrangement through regulation of phosphorylation of MLC. Furthermore, RSL3 increased 4HNE levels (Figure 6C), and 4HNE treatment of HULEC-5a cells induced EC barrier disruption and increased stress fibers (Figure 6D–F), which is consistent with the previous findings, suggesting that RSL3-induced EC barrier disruption occurs through 4HNE-increased stress fiber formation. 

### 3.6. RSL3 at 200 nm Concentration Did Not Induce Cell Death

**Figure 7:**
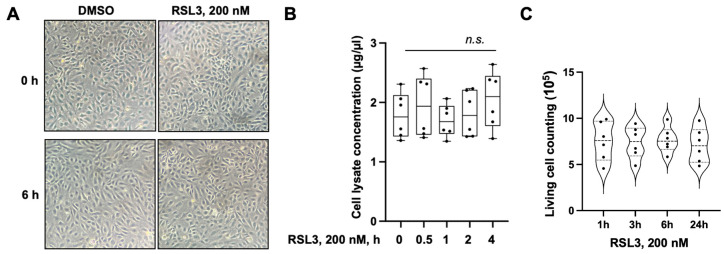
RSL3 (200 nM) did not induce cell death. (**A**) HULEC-5a cells were treated with RSL3 (200 nM) for 0 and 6 h. Cell shapes were captured using a light microscope. (**B**) HULEC-5a cells were treated with RSL3 (200 nM) for 0–4 h. Total cell lysates were analyzed (*n* = 6). No significant (n.s.) differences (*p* > 0.1) among all the groups, as determined via two-way ANOVA. (**C**) HULEC-5a cells were treated with RSL3 (200 nM) for 1, 3, 6, and 24 h. Live cell counting was performed using a Countess Automated Cell Counter (Invitrogen). There were no significant differences (*p* > 0.1) between the groups, as determined using two-way ANOVA.

RSL3 is an inducer of ferroptosis in various cell types, including ECs [21]. To determine if the effect of RSL3 at 100 and 200 nM on EC barrier function is dependent on cell death, we examined cell viability. We did not detect any changes regarding cell shape and confluency after RSL3 treatment for 6 h (Figure 7A). Total protein concentrations in the cell lysates from cells treated with RSL3 remained at similar levels compared to the cell lysates from control cells (Figure 7B). Live cell counts were not significantly altered by RSL3 200 nM up to 24 h (Figure 7C), indicating that RSL3 treatment at 200 nM does not induce cell death. Taken together, the effects of RSL3 on EC barrier and migration in this study are not due to cell death. This study reveals a new role of RSL3 in regulation of EC barrier integrity.

## 4. Discussion

Ferroptosis is a newly identified form of cell death that is caused by oxidative stress and lipid peroxidation [13]. RSL3 is an inhibitor of GPX4, and its effect on ferroptosis is dependent on its concentration and time of treatment [19,21]. The role of RSL3 in inducing cancer cell death has been well studied. In this study, we delineate that non-lethal doses of RSL3 modulate EC barrier integrity by diminishing S1P-induced barrier enhancement and increasing EC stress fiber formation. This study discovers new molecular mechanisms by which RSL3 diminishes EC functions.

S1PR1 is the main isoform of S1P receptors in ECs. S1P-mediated EC barrier enhancement has been shown to occur through ligation and activation of S1PR1 [8,29,30,31]. S1PR1 degradation has not been well studied. WWP2-mediated ubiquitination of S1PR1 promotes S1PR1 proteasomal degradation [32]. In this study, RSL3 at low dose (50 nM) induced S1PR1 degradation in the proteasome and lysosome. The ubiquitination, 4HNE modification, and oxidation of S1PR1 were not detected in the system, indicating that an unrevealed “degron” on S1PR1 regulates RSL3-induced S1PR1 degradation. In addition to ubiquitination and oxidation, other PTMs such as sumoylation and neddylation also regulate protein degradation. Phosphorylation and ubiquitination of S1PR1 have been reported [30,32], while future studies are needed to determine other PTMs and their roles in regulation of S1PR1-mediated EC function. We show that inhibition of lipid rafts attenuated S1PR1 degradation, suggesting that S1PR1 internalization is essential for RSL3-induced S1PR1 degradation. In addition to GPX4, RSL3 has been shown to target other proteins, such as selenoprotein T and protein SMG8 [19]. Thus, RSL3-induced S1PR1 degradation occurs through a PTM of S1PR1-independent manner. We hypothesize that RSL3 may target or modify one of the S1PR1-binding proteins and induce complex internalization and degradation. S1PR1 degradation has been shown to modulate immunosuppression [41]. A non-lethal dose of RSL3 may be used as an immunosuppressant in organ transplantation or immune diseases. 

Cytoskeletal rearrangement in ECs is induced by edemagenic agonists, such as thrombin, lysophosphatidic acid (LPA), and LPS [9,34,38]. MLC phosphorylation is an essential step for actomyosin activation and stress fiber formation. Protein kinases, such as MLCK and ROCK, phosphorylate MLC, while phosphatase MLCP reverses the process [33,34]. Increases in MLC phosphorylation can be achieved via the activation of kinases and the inhibition of phosphatase. In this study, RSL3 at non-lethal doses (100 and 200 nM) induced stress fiber formation and phosphorylation of MLC, suggesting that RSL3-induced EC hyperpermeability occurs through increases in cytoskeletal rearrangement. Activation of p38 MAPK has been shown to trigger EC barrier disruption [42,43]. We found that RSL3 also activated p38 MAPK. The molecular mechanisms by which RSL3 induces phosphorylation of MLC and p38 MAPK have not been revealed. RSL3 increases 4HNE production, and this is also observed in our system. Moreover, 4HNE has been shown to increase ROS production, phosphorylation of p38 MAPK, and cytoskeletal rearrangement and to reduce EC barrier integrity [44,45]. It is likely that the effect of a non-lethal level of RSL3 on EC barrier disruption is dependent on its 4HNE production. In the future study, the role of GPX4 activation in EC barrier function needs to be further investigated. EC inflammation regulates immune cell adhesion to ECs. The effect of RSL3 on EC inflammatory responses, such as ICAM1 and VCAM1 expression, has not been investigated. Given that RSL3 induces phosphorylation of p38 MAPK, we predict that RSL3 may increase EC inflammation. Future studies will investigate the effect of non-lethal doses of RSL3 on EC inflammation. 

## 5. Conclusions

**Figure 8:**
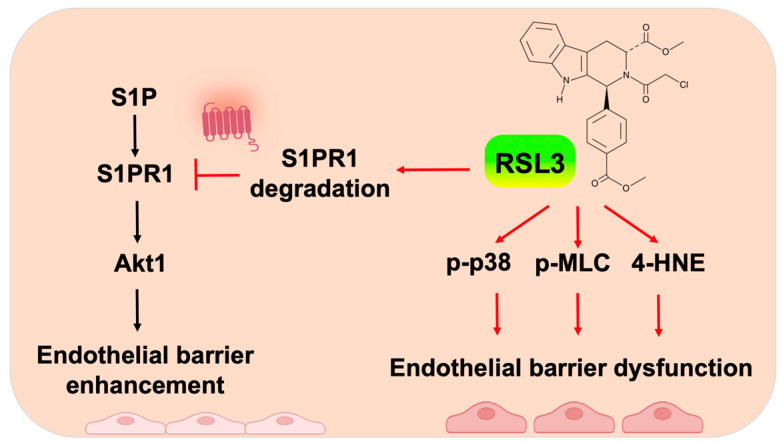
Non-lethal RSL3 reduces EC barrier integrity through degradation of S1PR1 and promotion of cytoskeletal rearrangement.

In this study, we delineate that non-lethal RSL3 reduces EC barrier integrity through degradation of S1PR1 and promotion of cytoskeletal rearrangement (Figure 8). Our findings indicate a detrimental effect of RSL3 in EC barrier integrity in a ferroptosis-independent manner. This study provides a platform for further investigating the molecular mechanisms by which RSL3 mediates EC dysfunction in vascular diseases. The data raises a caution that even non-lethal RSL3 may induce detrimental effects in EC barrier integrity. 

## Data Availability

All the materials and methods are available upon request for academic research purposes.
